# Supplementary material for: Chemical engineering of therapeutic siRNAs for allele-specific gene silencing in Huntington’s disease models
Source: Nat Commun. 2022 Oct 3;13:5802. doi: 10.1038/s41467-022-33061-x (PMC9530163; doi:10.1038/s41467-022-33061-x)
Supplement: Supplementary file 1 — Supplementary Information [file 41467_2022_33061_MOESM1_ESM.pdf]

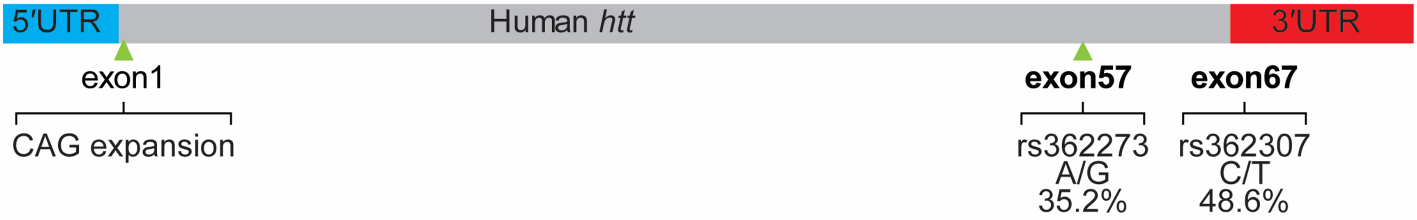

Supplementary Figure 1. A simplified map of the human *htt* gene shows regions of interest. Exons which are highlighted in bold contain SNPs with high rates of heterozygosity, used for subsequent experiments. 35.2% of HD patients are heterozygous (A/G) at rs362273, and 48.6% of HD patients are heterozygous (C/T) at rs362307.

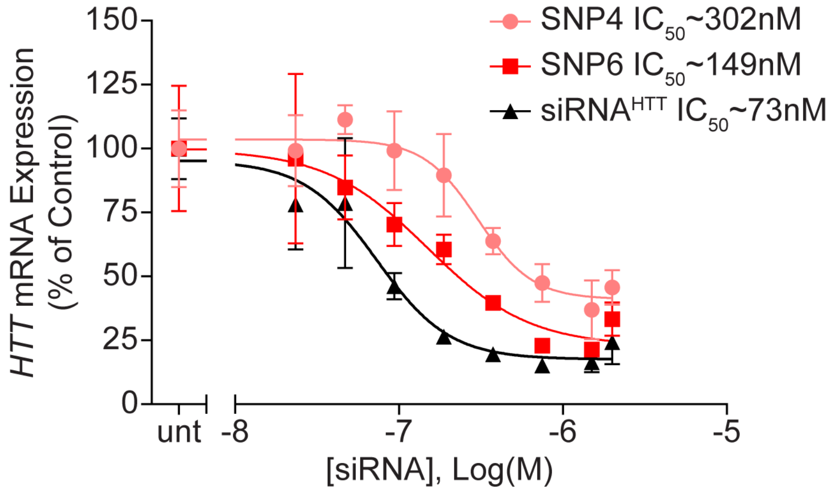

Supplementary Figure 2. **SNP-targeting siRNAs silence htt mRNA with a moderate reduction in potency compared to pan-HTT targeting siRNAs.** HeLa cells were treated with siRNAs via passive uptake for 72 hours. Endogenous huntingtin mRNA levels were measured with Quantigene 2.0 assay and normalized to PPIB; n=3 wells/treatment. Data are presented as mean ± SD. Source data are provided as a source data file.

a.

6-11A guide 5’-P(mU)#(fU)#(mC)(fU)(mG)(**fU**)(mA)(fG)(mC)(fA)(**mG**)(fC)(mA)#(fG)#(mC)#(fU)#(mU)#(fC)#(mU)#(fC)
Single mismatch 3’-(rA)(rG)(rA)(rC)(**rG**)(rU)(rC)(rG)(rU)(rC)(rG)(rU)(rC)(rG)(rA)(rA)(rG)(rA)(rG)-5’

6-11A guide 5’-P(mU)#(fU)#(mC)(fU)(mG)(**fU**)(mA)(fG)(mC)(fA)(**mG**)(fC)(mA)#(fG)#(mC)#(fU)#(mU)#(fC)#(mU)#(fC)
Full match 3’-(rA)(rG)(rA)(rC)(**rA**)(rU)(rC)(rG)(rU)(rC)(rG)(rU)(rC)(rG)(rA)(rA)(rG)(rA)(rG)-5’

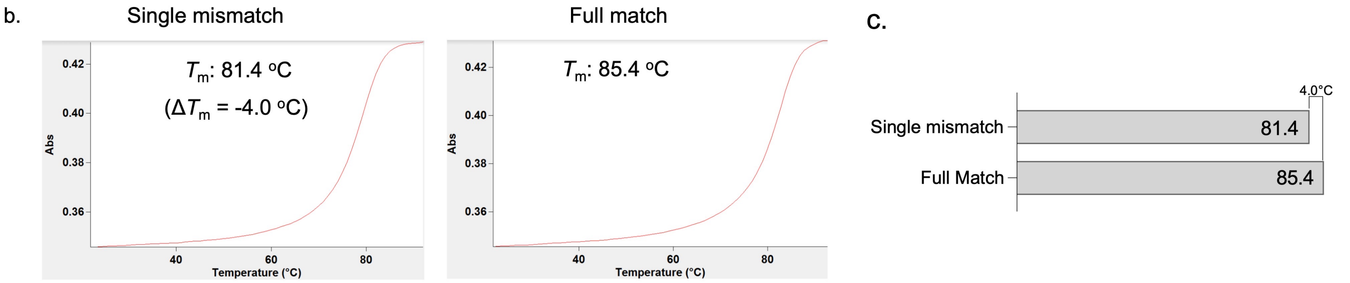

d.

6-11A guide 5’-P(mU)#(fU)#(mC)(fU)(mG)(**fU**)(mA)(fG)(mC)(fA)(**mG**)(fC)(mA)#(fG)#(mC)#(fU)#(mU)#(fC)#(mU)#(fC)
Single mismatch 3’-(rA)(rG)(rA)(rC)(**rG**)(rU)(rC)(rG)(rU)(rC)(rG)(rU)(rC)-5’

6-11A guide 5’-P(mU)#(fU)#(mC)(fU)(mG)(**fU**)(mA)(fG)(mC)(fA)(**mG**)(fC)(mA)#(fG)#(mC)#(fU)#(mU)#(fC)#(mU)#(fC)
Full match 3’-(rA)(rG)(rA)(rC)(**rA**)(rU)(rC)(rG)(rU)(rC)(rG)(rU)(rC)-5’

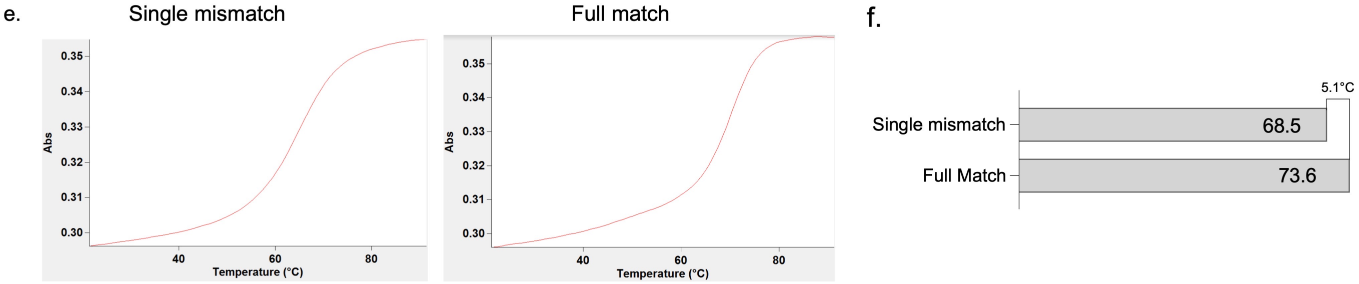

***Supplementary Figure 3*: A single mismatch in the seed region of the siRNA guide strand does not have a significant impact on duplex stability.** Guide strands for SNP6-11 were hybridized to 19 nucleotide and 13 nucleotide RNA strands, with or without a mismatch in the seed, and melting temperature was measured by thermo stability assay. **(a,d)** Sequence and structureofthe 6-11 guide strand and a complementary 19 nucleotide RNA or 13 nucleotide RNA. **(b,e)** Tm curves for SNP6-11 hybridized to a 19mer complementary RNA or 13mer complementary RNA strand, with a single mismatch or full matched sequence. **(c,f**) Graph comparing melting temperatures of the full match sequence with a single mismatch included, exhibiting a minimal change in melting temperature, whether hybridized to a 13nt or 19nt complementary RNA strand.

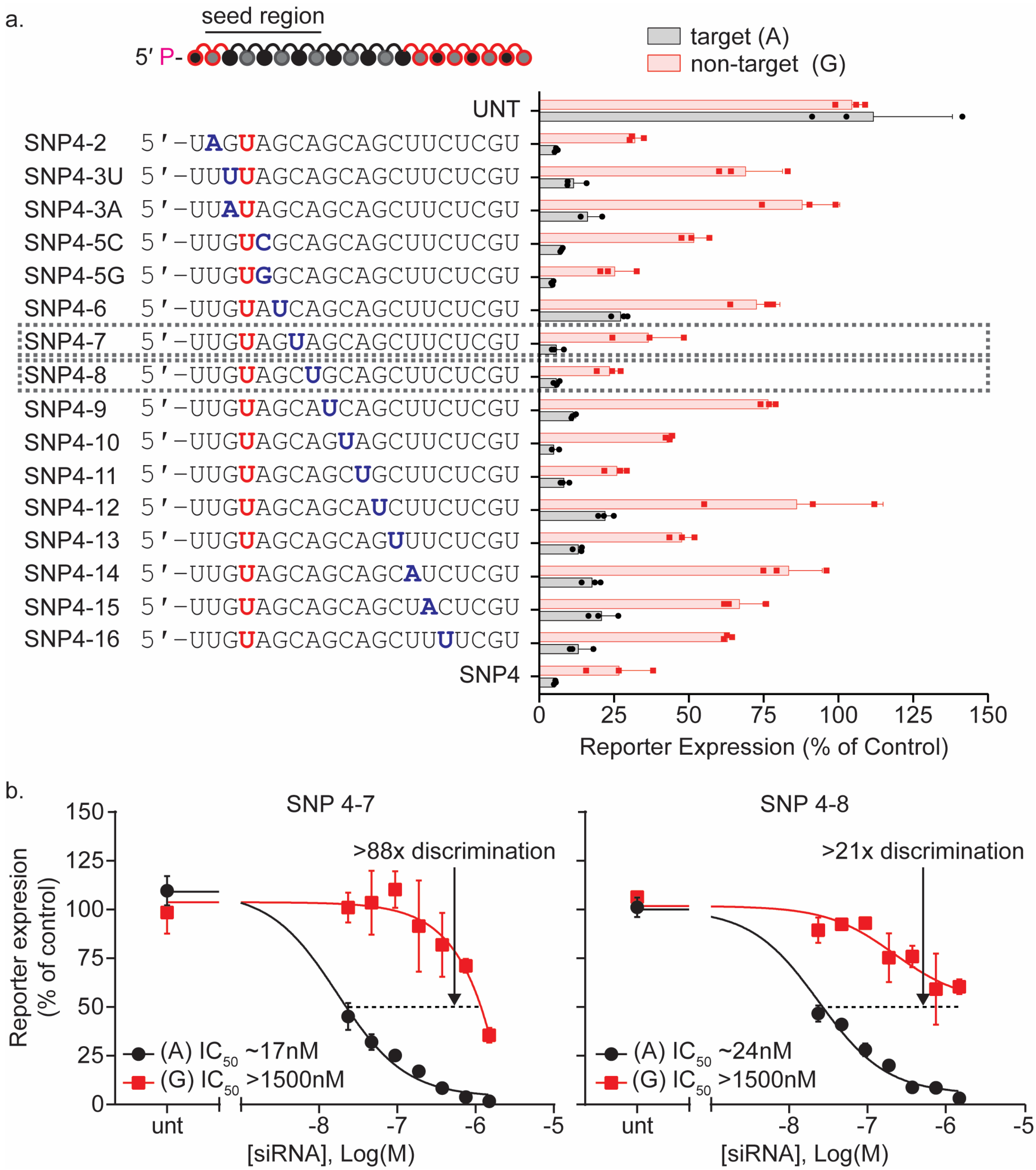

***Supplementary Figure 4*. Addition of secondary mismatch is well tolerated in target silencing but reduces non-target activity. (a)** siRNA SNP4 (from primary screen; main Fig. 1) was screened for introduction of secondary mismatch, resulting in increased discrimination; n=3 wells/treatment. **(b)** Dual-luciferase reporter assay dose response of lead compounds identifies SNP4-7 as the best-performing siRNA, improving discrimination more than 10x when compared to SNP4 (main Figure 1c); n=2 wells/treatment. All data are presented as mean ± SD. Source data are provided as a source data file.

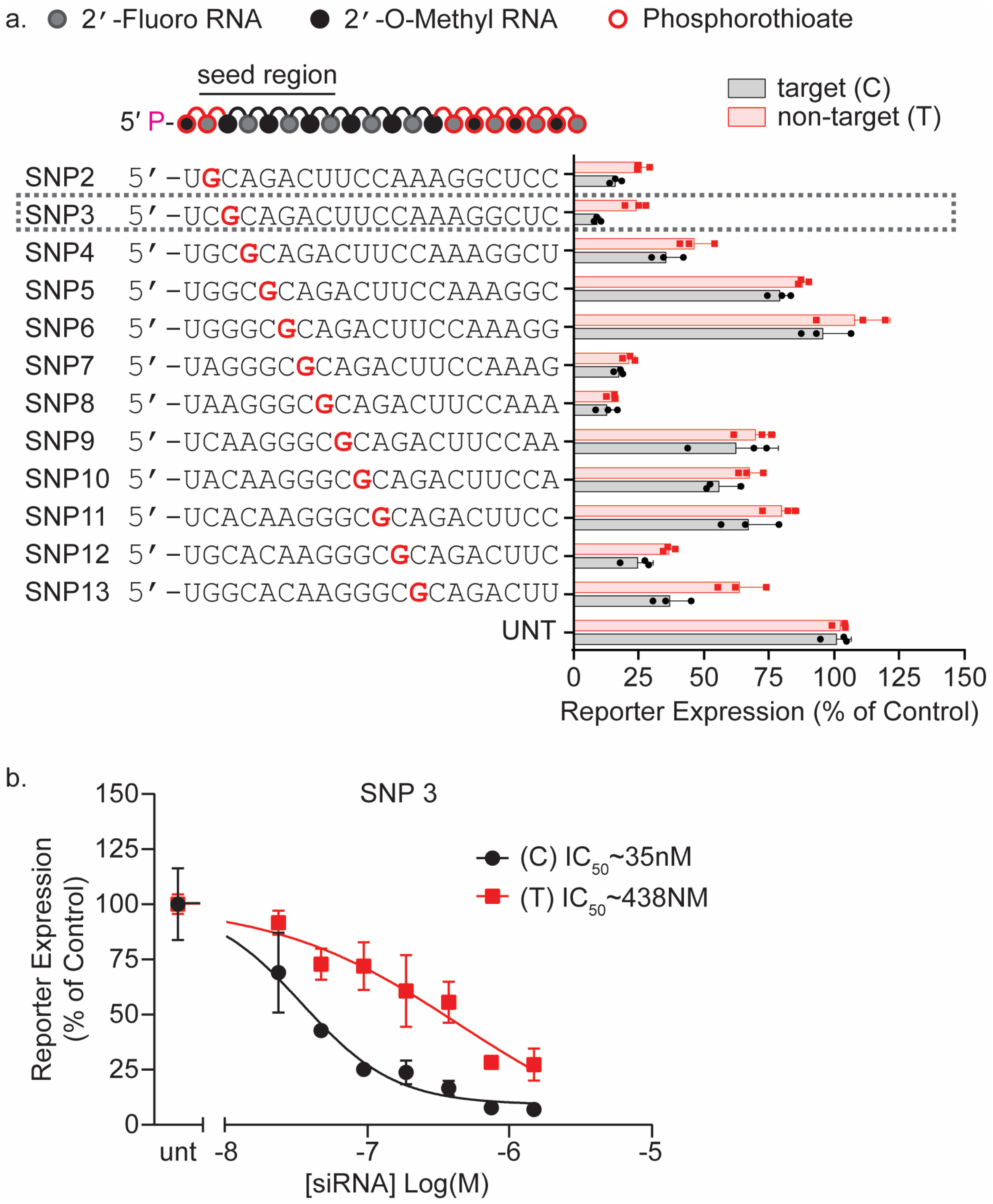

a.
2′-Fluoro RNA
2′-O-Methyl RNA
Phosphorothioate
seed region
5′ P-
target (C)
non-target (T)
SNP2 5′-UGCAGACUUCCAAAGGCUCC
SNP3 5′-UCGCAGACUUCCAAAGGCUC
SNP4 5′-UGCGCAGACUUCCAAAGGCU
SNP5 5′-UGGCGCAGACUUCCAAAGGC
SNP6 5′-UGGGCGCAGACUUCCAAAGG
SNP7 5′-UAGGGCGCAGACUUCCAAAG
SNP8 5′-UAAGGGCGCAGACUUCCAAA
SNP9 5′-UCAAGGGCGCAGACUUCCAA
SNP10 5′-UACAAGGGCGCAGACUUCCA
SNP11 5′-UCACAAGGGCGCAGACUUCC
SNP12 5′-UGCACAAGGGCGCAGACUUC
SNP13 5′-UGGCACAAGGGCGCAGACUU
UNT
0 25 50 75 100 125 150
Reporter Expression (% of Control)
b.
SNP 3
150 125 100 75 50 25 0
Reporter Expression (% of Control)
(C) IC50~35nM
(T) IC50~438NM
unt -8 -7 -6 -5
[siRNA] Log(M)

Supplementary Figure 5. **Screening method for sequence and mismatch selection can be used to produce effective SNP-selective siRNAs for a different SNP site. (a)** Results of primary screen finds optimal sequences for targeting SNP site rs362307 (highlighted in red). Compounds were tested using a dual-luciferase reporter assay system in HeLa cells. The (psiCheck) reporter plasmids contain a 40mer region of huntingtin, including the target SNP (C) (black), and non-target (U) isoform (red). Cells were treated for 72 hours at 1.5μM of siRNA. A panel of siRNA sequences were synthesized in a cholesterol-conjugated scaffold with phosphorothioate and alternating 2′-F and 2′-OMe backbone modifications. By walking the siRNA sequence around SNP site rs362307, we find multiple compounds with varying degrees of efficacy and discrimination; n=3 wells/treatment. **(b)** Dose response of SNP3, which was selected for further screening of secondary mismatches; n=3 wells/treatment. All data are presented as mean ± SD. Source data are provided as a source data file.

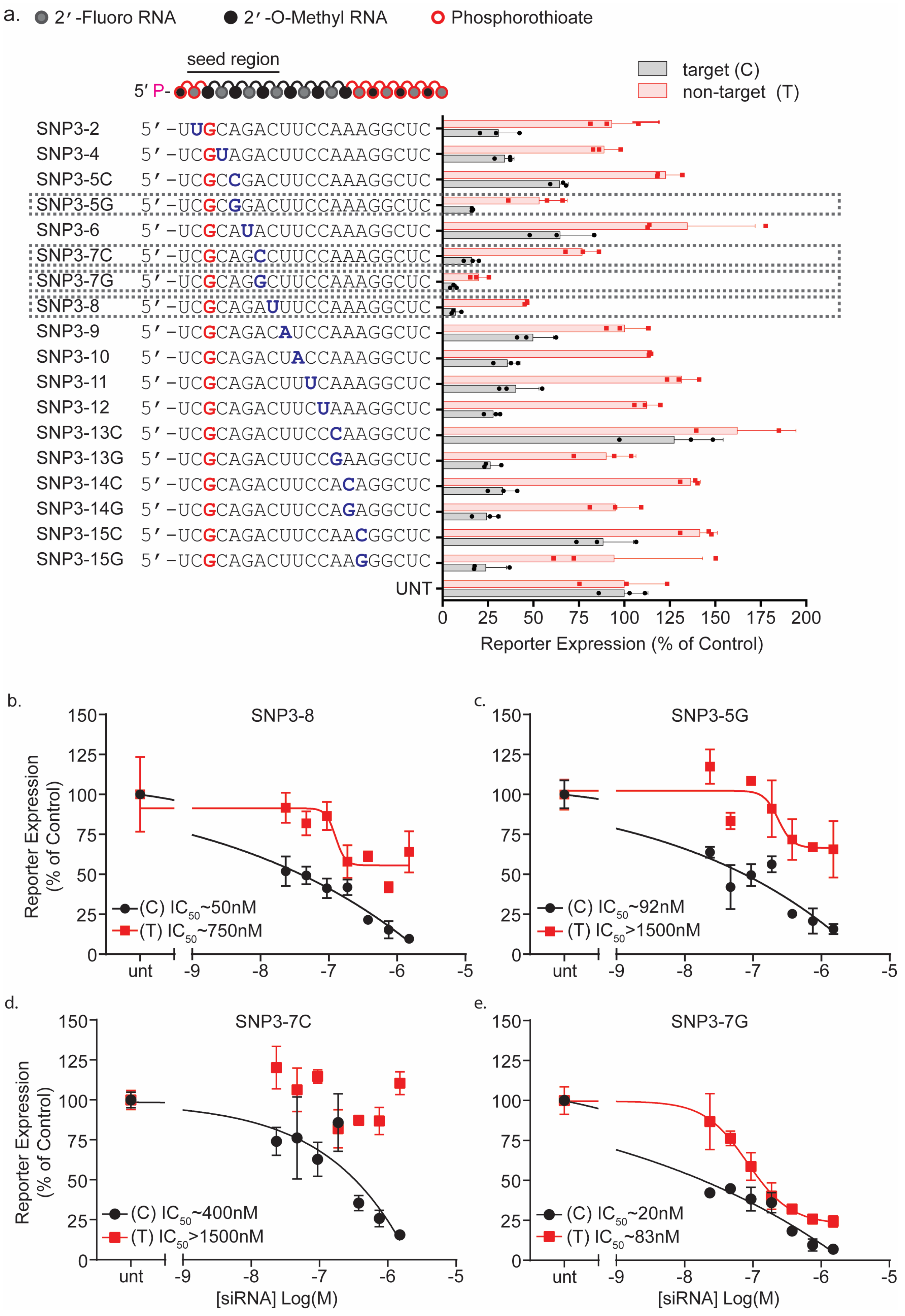

a.
2′-Fluoro RNA
2′-O-Methyl RNA
Phosphorothioate
seed region
5′ P-
target (C)
non-target (T)
SNP3-2 5′-UUGCAGACUUCCAAAGGCUC
SNP3-4 5′-UCGUAGACUUCCAAAGGCUC
SNP3-5C 5′-UCGCCGACUUCCAAAGGCUC
SNP3-5G 5′-UCGCGGACUUCCAAAGGCUC
SNP3-6 5′-UCGCAUACUUCCAAAGGCUC
SNP3-7C 5′-UCGCAGCCUUCCAAAGGCUC
SNP3-7G 5′-UCGCAGGCUUCCAAAGGCUC
SNP3-8 5′-UCGCAGAUUUCCAAAGGCUC
SNP3-9 5′-UCGCAGACAUCCAAAGGCUC
SNP3-10 5′-UCGCAGACUACCAAAGGCUC
SNP3-11 5′-UCGCAGACUUUCAAAGGCUC
SNP3-12 5′-UCGCAGACUUCUAAAGGCUC
SNP3-13C 5′-UCGCAGACUUCCCAAGGCUC
SNP3-13G 5′-UCGCAGACUUCCGAAGGCUC
SNP3-14C 5′-UCGCAGACUUCCACAGGCUC
SNP3-14G 5′-UCGCAGACUUCCAGAGGCUC
SNP3-15C 5′-UCGCAGACUUCCAACGGCUC
SNP3-15G 5′-UCGCAGACUUCCAAGGGCUC
UNT
0 25 50 75 100 125 150 175 200
Reporter Expression (% of Control)
b.
SNP3-8
(C) IC50~50nM
(T) IC50~750nM
c.
SNP3-5G
(C) IC50~92nM
(T) IC50>1500nM
d.
SNP3-7C
(C) IC50~400nM
(T) IC50>1500nM
e.
SNP3-7G
(C) IC50~20nM
(T) IC50~83nM
Reporter Expression (% of Control)
0 25 50 75 100 125 150
unt -9 -8 -7 -6 -5
[siRNA] Log(M)

***Supplementary Figure 6*. Optimization of the lead compound targeting rs362307 increases discrimination while retaining potency.** SNP3 was selected for further optimization **(a)** Introduction of a secondary mismatch to positions 5 and 7 increases allelic discrimination; n=3 wells/treatment. **(b-e)** A dose response of lead secondary-mismatch siRNAs validates an increase in discrimination based on the position of the mismatch. A C:U wobble mismatch at position 7 (SNP3-7C; **d**) results in a decrease in non-target activity when compared to a G:U mismatch at the same position (SNP3-7G; **e**); n=3 wells/treatment group. All data are presented as mean ± SD. Source data are provided as a source data file.

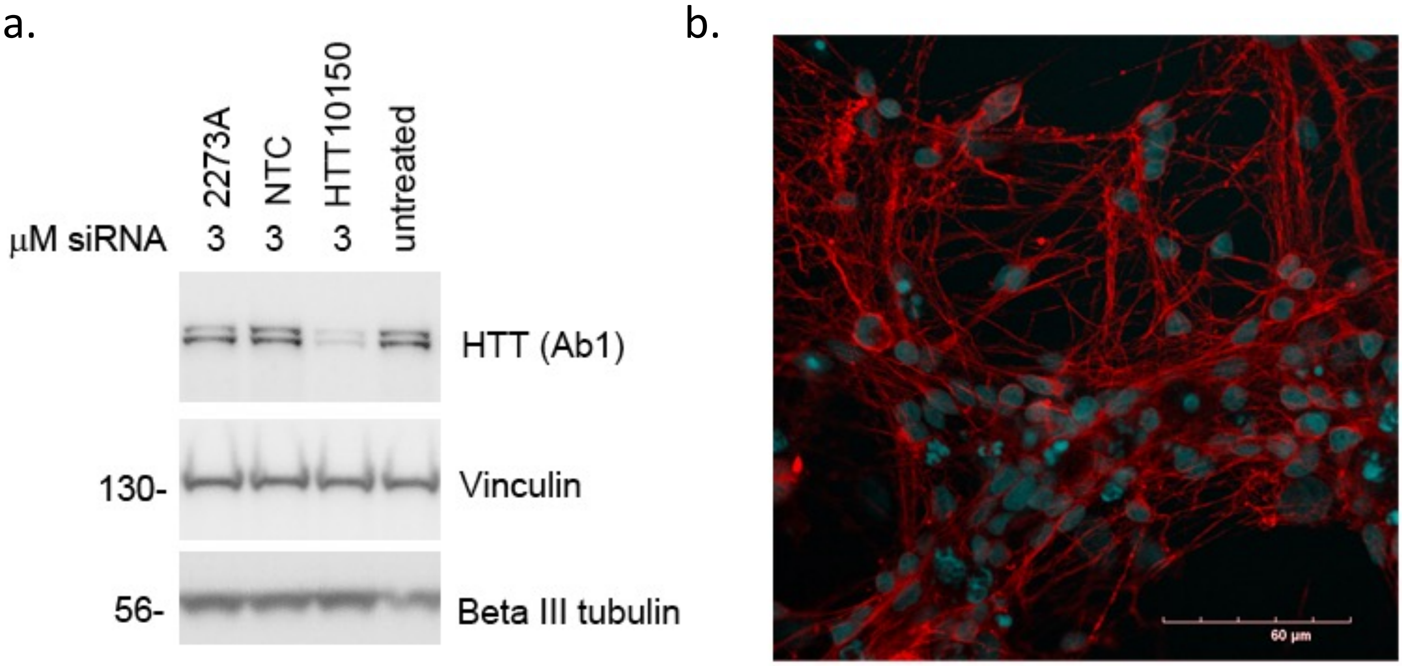

Supplementary Figure 7. **Human HD109 neuron cultures show a high percentage of neurons. (a)** Western blot analysis of HD109 NSCs, which are heterozygous at SNP2273, showed lowering of mutant HTT (slower migrating band) compared to wild-type HTT (faster migrating band) with SNP2273A. For siRNA dosing regimen, see Methods. Equal protein (10 μg) were loaded per lane. Blots were probed with anti-HTT antibody Ab1 and vinculin as a housekeeping protein and the neuronal marker βIII tubulin. Experiments were performed with n=10 samples per group. For full blots, see also source data for figures 5a and 5b. **(b)** Confocal immunofluorescent image of neuronal cultures stained for the neuronal marker βIII tubulin (Red) and the nuclear marker Hoechst (Blue) show high percentage of neurons in the cultures. Scale Bar=60mm; 40X objective. The micrograph is a representative field from 2 cover slips.

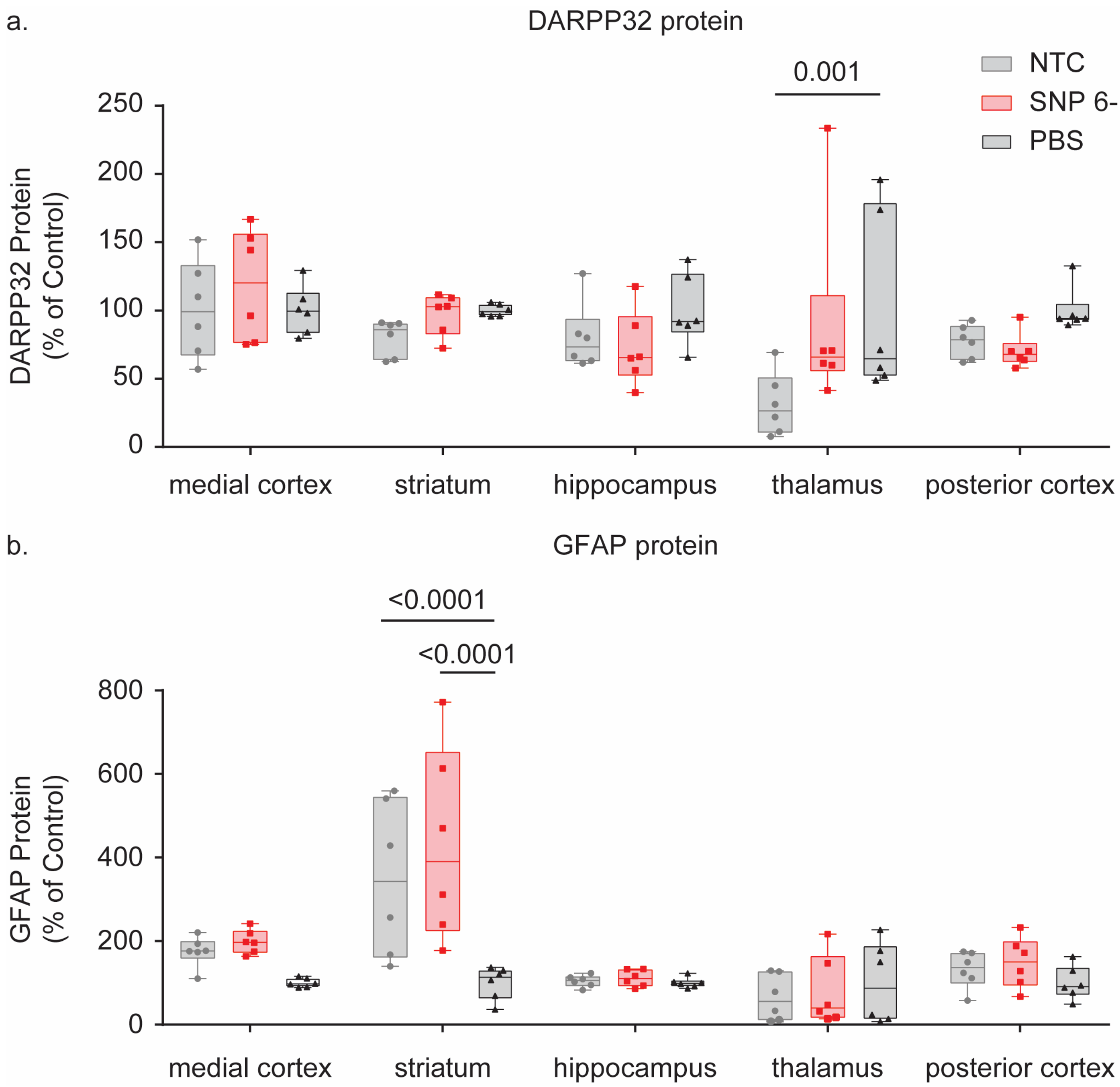

Supplementary Figure 8. **Selective silencing of mutant *HTT* is well tolerated. (a)** SNP 6-11 siRNA has no impact on DARPP32 levels in treated mice although a decrease in DARPP-32 was observed in the thalamus of NTC-treated mice. **(b)** GFAP levels were also not affected by siRNA treatment, except in the striatum where an increase in GFAP was observed in both siRNA groups. A two-way ANOVA with multiple comparisons was performed for all protein analysis, comparing treatment groups to the PBS control for each brain region; n=6 animals per group. Error bars extend to minimum and maximum value. (ProteinSimple) The lower bound of the box is the 25th percentile and the upper is the 75th. The line represents the median. Source data are provided as a source data file.

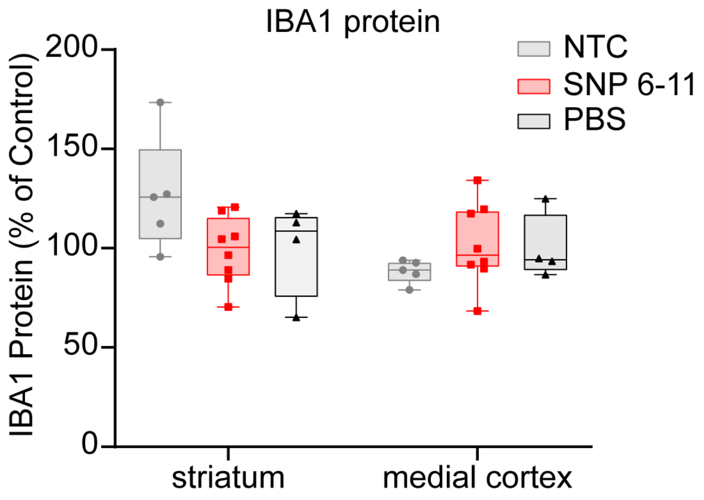

Supplementary Figure 9. **SNP6-11 brain administration does not elicit microglial activation as indicated by lack of IBA1 elevation.** BAC-HD mice were injected with 225 µg of SNP6-11, NTC siRNAs and PBS. Levels of IBA1 expression in striatum and medial cortex, evaluated by automated western blot and normalized to GAPDH loading control. N=5-6, one-way ANOVA with Tukey multiple comparison correction; n=3 biological replicates per group. Error bars extend to minimum and maximum value. The lower bound of the box is the 25th percentile and the upper is the 75th. The line represents the median. Source data are provided as a source data file.

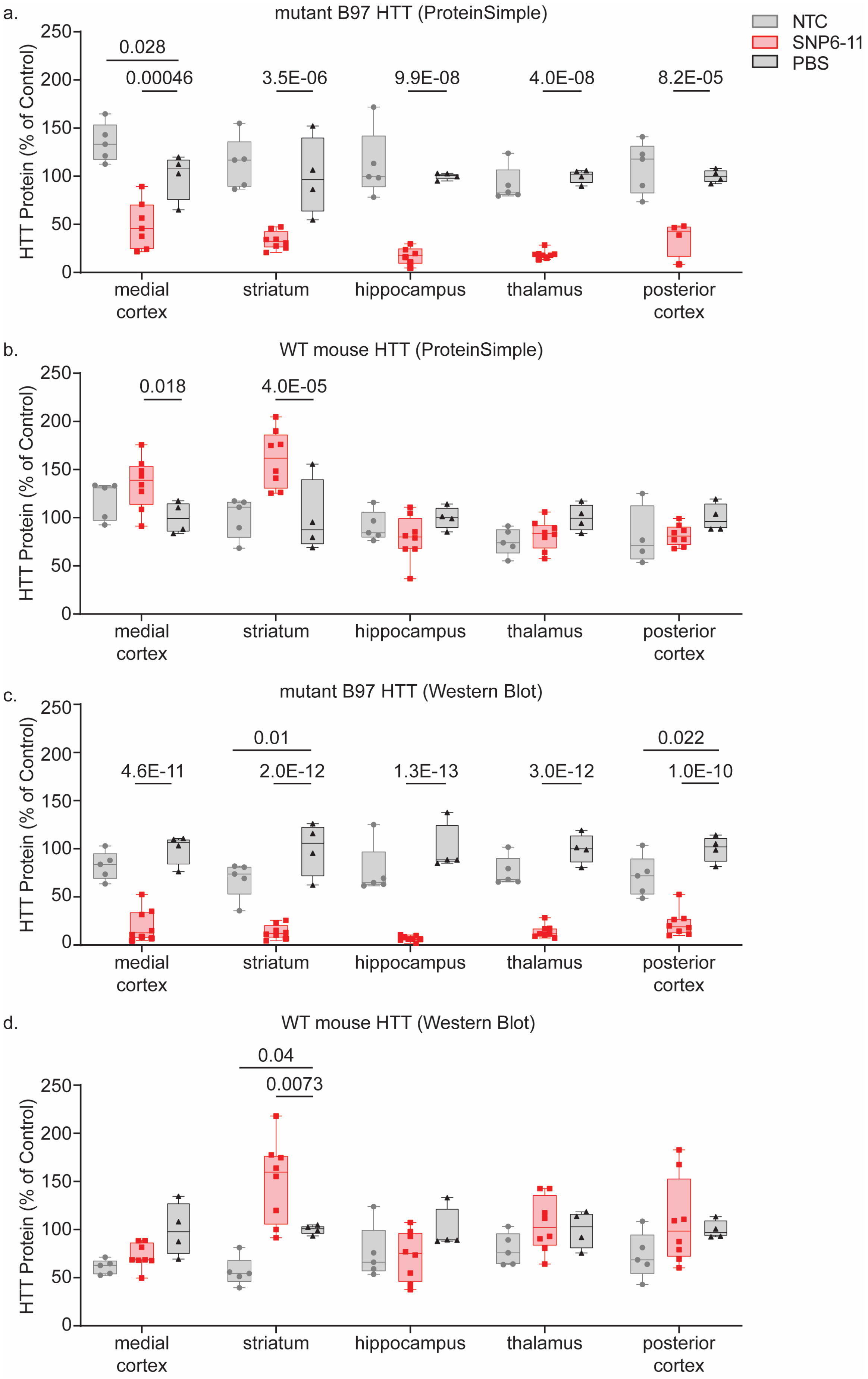
a.
mutant B97 HTT (ProteinSimple)
NTC
SNP6-11
PBS
HTT Protein (% of Control)
0.028
0.00046
3.5E-06
9.9E-08
4.0E-08
8.2E-05
medial cortex
striatum
hippocampus
thalamus
posterior cortex
b.
WT mouse HTT (ProteinSimple)
0.018
4.0E-05
c.
mutant B97 HTT (Western Blot)
0.01
0.022
4.6E-11
2.0E-12
1.3E-13
3.0E-12
1.0E-10
d.
WT mouse HTT (Western Blot)
0.04
0.0073

Supplementary Figure 10. **Increasing siRNA dose to 40nmols via ICV injection does not increase silencing of the HTT protein, or allelic discrimination. (a)** When treated with 450μg (20nmol, 10nmol/side) siRNA SNP6-11, selective silencing of mutant HTT protein (measured by WES Protein Simple) is achieved. There is an increase in wild-type HTT expression in the striatum and medial cortex compared to the controls, which is not seen at a 10nmol dose or in human cells. **(b)** Western blot shows that results are consistent among assays, except for the medial cortex which shows an increase in WT HTT in the PBS control. A two-way ANOVA with multiple comparisons was performed for all protein analysis, comparing treatment groups to PBS control for each brain region; n=3 animals per group. Error bars extend to minimum and maximum value. The lower bound of the box is the 25th percentile and the upper is the 75th. The line represents the median. Source data are provided as a source data file.

Supplementary Table 1. complete list of all sequences of all siRNAs used in this study, with corresponding *in vitro* IC50 values for compounds if a dose response was performed.

siRNA name	SNP position	additional mismatch position	antisense strand sequence	sense strand sequence	IC 50 (target) [nM]	IC50 (non-target) [nM]	Target SNP site	Related Figure (s)
SNP2-0	2	0	P(mU)#(fU)#(mA)(fG)(mC)(fA)(mG)(fC)(mA)(fG)(mC)(fU)(mU)#(fC)#(mU)#(fC)#(mG)#(fU)#(mG)#(fG)	(fA)#(mG)#(fA)(mA)(fG)(mC)(fU)(mG)(fC)(mU)(fG)(mC)(fU)#(mA)#(fA)-TegChol			rs362273	1
SNP3-0	3	0	P(mU)#(fG)#(mU)(fA)(mG)(fC)(mA)(fG)(mC)(fA)(mG)(fC)(mU)#(fU)#(mC)#(fU)#(mC)#(fG)#(mU)#(fG)	(fG)#(mA)#(fA)(mG)(fC)(mU)(fG)(mC)(fU)(mG)(fC)(mU)(fA)#(mC)#(fA)-TegChol			rs362273	1
SNP4-0	4	0	P(mU)#(fU)#(mG)(fU)(mA)(fG)(mC)(fA)(mG)(fC)(mA)(fG)(mC)#(fU)#(mU)#(fC)#(mU)#(fC)#(mG)#(fU)	(fA)#(mA)#(fG)(mC)(fU)(mG)(fC)(mU)(fG)(mC)(fU)(mA)(fC)#(mA)#(fA)-TegChol	38	803	rs362273	1,S1
SNP4-2	4	2	P(mU)#(fA)#(mG)(fU)(mA)(fG)(mC)(fA)(mG)(fC)(mA)(fG)(mC)#(fU)#(mU)#(fC)#(mU)#(fC)#(mG)#(fU)	(fA)#(mA)#(fG)(mC)(fU)(mG)(fC)(mU)(fG)(mC)(fU)(mA)(fC)#(mA)#(fA)-TegChol			rs362273	S4
SNP4-3U	4	3	P(mU)#(fU)#(mU)(fU)(mA)(fG)(mC)(fA)(mG)(fC)(mA)(fG)(mC)#(fU)#(mU)#(fC)#(mU)#(fC)#(mG)#(fU)	(fA)#(mA)#(fG)(mC)(fU)(mG)(fC)(mU)(fG)(mC)(fU)(mA)(fC)#(mA)#(fA)-TegChol			rs362273	S4
SNP4-3A	4	3	P(mU)#(fU)#(mA)(fU)(mA)(fG)(mC)(fA)(mG)(fC)(mA)(fG)(mC)#(fU)#(mU)#(fC)#(mU)#(fC)#(mG)#(fU)	(fA)#(mA)#(fG)(mC)(fU)(mG)(fC)(mU)(fG)(mC)(fU)(mA)(fC)#(mA)#(fA)-TegChol			rs362273	S4
SNP4-5C	4	5	P(mU)#(fU)#(mG)(fU)(mC)(fG)(mC)(fA)(mG)(fC)(mA)(fG)(mC)#(fU)#(mU)#(fC)#(mU)#(fC)#(mG)#(fU)	(fA)#(mA)#(fG)(mC)(fU)(mG)(fC)(mU)(fG)(mC)(fU)(mA)(fC)#(mA)#(fA)-TegChol			rs362273	S4
SNP4-5G	4	5	P(mU)#(fU)#(mG)(fU)(mG)(fG)(mC)(fA)(mG)(fC)(mA)(fG)(mC)#(fU)#(mU)#(fC)#(mU)#(fC)#(mG)#(fU)	(fA)#(mA)#(fG)(mC)(fU)(mG)(fC)(mU)(fG)(mC)(fU)(mA)(fC)#(mA)#(fA)-TegChol			rs362273	S4
SNP4-6	4	6	P(mU)#(fU)#(mG)(fU)(mA)(fU)(mC)(fA)(mG)(fC)(mA)(fG)(mC)#(fU)#(mU)#(fC)#(mU)#(fC)#(mG)#(fU)	(fA)#(mA)#(fG)(mC)(fU)(mG)(fC)(mU)(fG)(mC)(fU)(mA)(fC)#(mA)#(fA)-TegChol			rs362273	S4
SNP4-7	4	7	P(mU)#(fU)#(mG)(fU)(mA)(fG)(mU)(fA)(mG)(fC)(mA)(fG)(mC)#(fU)#(mU)#(fC)#(mU)#(fC)#(mG)#(fU)	(fA)#(mA)#(fG)(mC)(fU)(mG)(fC)(mU)(fG)(mC)(fU)(mA)(fC)#(mA)#(fA)-TegChol	17	>5000	rs362273	S4
SNP4-8	4	8	P(mU)#(fU)#(mG)(fU)(mA)(fG)(mC)(fU)(mG)(fC)(mA)(fG)(mC)#(fU)#(mU)#(fC)#(mU)#(fC)#(mG)#(fU)	(fA)#(mA)#(fG)(mC)(fU)(mG)(fC)(mU)(fG)(mC)(fU)(mA)(fC)#(mA)#(fA)-TegChol	24	220	rs362273	S4
SNP4-9	4	9	P(mU)#(fU)#(mG)(fU)(mA)(fG)(mC)(fA)(mU)(fC)(mA)(fG)(mC)#(fU)#(mU)#(fC)#(mU)#(fC)#(mG)#(fU)	(fA)#(mA)#(fG)(mC)(fU)(mG)(fC)(mU)(fG)(mC)(fU)(mA)(fC)#(mA)#(fA)-TegChol			rs362273	S4
SNP4-10	4	10	P(mU)#(fU)#(mG)(fU)(mA)(fG)(mC)(fA)(mG)(fU)(mA)(fG)(mC)#(fU)#(mU)#(fC)#(mU)#(fC)#(mG)#(fU)	(fA)#(mA)#(fG)(mC)(fU)(mG)(fC)(mU)(fG)(mC)(fU)(mA)(fC)#(mA)#(fA)-TegChol			rs362273	S4
SNP4-11	4	11	P(mU)#(fU)#(mG)(fU)(mA)(fG)(mC)(fA)(mG)(fC)(mU)(fG)(mC)#(fU)#(mU)#(fC)#(mU)#(fC)#(mG)#(fU)	(fA)#(mA)#(fG)(mC)(fU)(mG)(fC)(mU)(fG)(mC)(fU)(mA)(fC)#(mA)#(fA)-TegChol			rs362273	S4
SNP4-12	4	12	P(mU)#(fU)#(mG)(fU)(mA)(fG)(mC)(fA)(mG)(fC)(mA)(fU)(mC)#(fU)#(mU)#(fC)#(mU)#(fC)#(mG)#(fU)	(fA)#(mA)#(fG)(mC)(fU)(mG)(fC)(mU)(fG)(mC)(fU)(mA)(fC)#(mA)#(fA)-TegChol			rs362273	S4
SNP4-13	4	13	P(mU)#(fU)#(mG)(fU)(mA)(fG)(mC)(fA)(mG)(fC)(mA)(fG)(mU)#(fU)#(mU)#(fC)#(mU)#(fC)#(mG)#(fU)	(fA)#(mA)#(fG)(mC)(fU)(mG)(fC)(mU)(fG)(mC)(fU)(mA)(fC)#(mA)#(fA)-TegChol			rs362273	S4
SNP4-14	4	14	P(mU)#(fU)#(mG)(fU)(mA)(fG)(mC)(fA)(mG)(fC)(mA)(fG)(mC)#(fA)#(mU)#(fC)#(mU)#(fC)#(mG)#(fU)	(fA)#(mA)#(fG)(mC)(fU)(mG)(fC)(mU)(fG)(mC)(fU)(mA)(fC)#(mA)#(fA)-TegChol			rs362273	S4
SNP4-15	4	15	P(mU)#(fU)#(mG)(fU)(mA)(fG)(mC)(fA)(mG)(fC)(mA)(fG)(mC)#(fU)#(mA)#(fC)#(mU)#(fC)#(mG)#(fU)	(fA)#(mA)#(fG)(mC)(fU)(mG)(fC)(mU)(fG)(mC)(fU)(mA)(fC)#(mA)#(fA)-TegChol			rs362273	S4
SNP4-16	4	16	P(mU)#(fU)#(mG)(fU)(mA)(fG)(mC)(fA)(mG)(fC)(mA)(fG)(mC)#(fU)#(mU)#(fU)#(mU)#(fC)#(mG)#(fU)	(fA)#(mA)#(fG)(mC)(fU)(mG)(fC)(mU)(fG)(mC)(fU)(mA)(fC)#(mA)#(fA)-TegChol			rs362273	S4
SNP5-0	5	0	P(mU)#(fC)#(mU)(fG)(mU)(fA)(mG)(fC)(mA)(fG)(mC)(fA)(mG)#(fC)#(mU)#(fU)#(mC)#(fU)#(mC)#(fG)	(fA)#(mG)#(fC)(mU)(fG)(mC)(fU)(mG)(fC)(mU)(fA)(mC)(fA)#(mG)#(fA)-TegChol			rs362273	1
SNP6-0	6	0	P(mU)#(fU)#(mC)(fU)(mG)(fU)(mA)(fG)(mC)(fA)(mG)(fC)(mA)#(fG)#(mC)#(fU)#(mU)#(fC)#(mU)#(fC)	(fG)#(mC)#(fU)(mG)(fC)(mU)(fG)(mC)(fU)(mA)(fC)(mA)(fG)#(mA)#(fA)-TegChol	24	174	rs362273	1,S1,2
SNP6-2	6	2	P(mU)#(fA)#(mC)(fU)(mG)(fU)(mA)(fG)(mC)(fA)(mG)(fC)(mA)#(fG)#(mC)#(fU)#(mU)#(fC)#(mU)#(fC)	(fG)#(mC)#(fU)(mG)(fC)(mU)(fG)(mC)(fU)(mA)(fC)(mA)(fG)#(mA)#(fA)-TegChol			rs362273	2
SNP6-3	6	3	P(mU)#(fU)#(mU)(fU)(mG)(fU)(mA)(fG)(mC)(fA)(mG)(fC)(mA)#(fG)#(mC)#(fU)#(mU)#(fC)#(mU)#(fC)	(fG)#(mC)#(fU)(mG)(fC)(mU)(fG)(mC)(fU)(mA)(fC)(mA)(fG)#(mA)#(fA)-TegChol			rs362273	2
SNP6-4	6	4	P(mU)#(fU)#(mC)(fA)(mG)(fU)(mA)(fG)(mC)(fA)(mG)(fC)(mA)#(fG)#(mC)#(fU)#(mU)#(fC)#(mU)#(fC)	(fG)#(mC)#(fU)(mG)(fC)(mU)(fG)(mC)(fU)(mA)(fC)(mA)(fG)#(mA)#(fA)-TegChol			rs362273	2
SNP6-5U	6	5	P(mU)#(fU)#(mC)(fU)(mU)(fU)(mA)(fG)(mC)(fA)(mG)(fC)(mA)#(fG)#(mC)#(fU)#(mU)#(fC)#(mU)#(fC)	(fG)#(mC)#(fU)(mG)(fC)(mU)(fG)(mC)(fU)(mA)(fC)(mA)(fG)#(mA)#(fA)-TegChol			rs362273	2
SNP6-5A	6	5	P(mU)#(fU)#(mC)(fU)(mA)(fU)(mA)(fG)(mC)(fA)(mG)(fC)(mA)#(fG)#(mC)#(fU)#(mU)#(fC)#(mU)#(fC)	(fG)#(mC)#(fU)(mG)(fC)(mU)(fG)(mC)(fU)(mA)(fC)(mA)(fG)#(mA)#(fA)-TegChol			rs362273	2
SNP6-7C	6	7	P(mU)#(fU)#(mC)(fU)(mG)(fU)(mC)(fG)(mC)(fA)(mG)(fC)(mA)#(fG)#(mC)#(fU)#(mU)#(fC)#(mU)#(fC)	(fG)#(mC)#(fU)(mG)(fC)(mU)(fG)(mC)(fU)(mA)(fC)(mA)(fG)#(mA)#(fA)-TegChol			rs362273	2
SNP6-7G	6	7	P(mU)#(fU)#(mC)(fU)(mG)(fU)(mG)(fG)(mC)(fA)(mG)(fC)(mA)#(fG)#(mC)#(fU)#(mU)#(fC)#(mU)#(fC)	(fG)#(mC)#(fU)(mG)(fC)(mU)(fG)(mC)(fU)(mA)(fC)(mA)(fG)#(mA)#(fA)-TegChol			rs362273	2
SNP6-8	6	8	P(mU)#(fU)#(mC)(fU)(mG)(fU)(mA)(fU)(mC)(fA)(mG)(fC)(mA)#(fG)#(mC)#(fU)#(mU)#(fC)#(mU)#(fC)	(fG)#(mC)#(fU)(mG)(fC)(mU)(fG)(mC)(fU)(mA)(fC)(mA)(fG)#(mA)#(fA)-TegChol			rs362273	2
SNP6-9	6	9	P(mU)#(fU)#(mC)(fU)(mG)(fU)(mA)(fG)(mU)(fA)(mG)(fC)(mA)#(fG)#(mC)#(fU)#(mU)#(fC)#(mU)#(fC)	(fG)#(mC)#(fU)(mG)(fC)(mU)(fG)(mC)(fU)(mA)(fC)(mA)(fG)#(mA)#(fA)-TegChol			rs362273	2
SNP6-10	6	10	P(mU)#(fU)#(mC)(fU)(mG)(fU)(mA)(fG)(mC)(fU)(mG)(fC)(mA)#(fG)#(mC)#(fU)#(mU)#(fC)#(mU)#(fC)	(fG)#(mC)#(fU)(mG)(fC)(mU)(fG)(mC)(fU)(mA)(fC)(mA)(fG)#(mA)#(fA)-TegChol			rs362273	2
SNP6-11 (G)	6	11	P(mU)#(fU)#(mC)(fU)(mG)(fC)(mA)(fG)(mC)(fA)(mU)(fC)(mA)#(fG)#(mC)#(fU)#(mU)#(fC)#(mU)#(fC)	(fG)#(mC)#(fU)(mG)(fC)(mU)(fG)(mC)(fU)(mG)(fC)(mA)(fG)#(mA)#(fA)-TegChol			rs362273	2,4,S3
SNP6-11 (A)	6	11	P(mU)#(fU)#(mC)(fU)(mG)(fU)(mA)(fG)(mC)(fA)(mU)(fC)(mA)#(fG)#(mC)#(fU)#(mU)#(fC)#(mU)#(fC)	(fG)#(mC)#(fU)(mG)(fC)(mU)(fG)(mC)(fU)(mA)(fC)(mA)(fG)#(mA)#(fA)-TegChol	28	>4500	rs362273	2,3,4,S3
SNP6-12	6	12	P(mU)#(fU)#(mC)(fU)(mG)(fU)(mA)(fG)(mC)(fA)(mG)(fU)(mA)#(fG)#(mC)#(fU)#(mU)#(fC)#(mU)#(fC)	(fG)#(mC)#(fU)(mG)(fC)(mU)(fG)(mC)(fU)(mA)(fC)(mA)(fG)#(mA)#(fA)-TegChol	24	>1000	rs362273	2
SNP6-13	6	13	P(mU)#(fU)#(mC)(fU)(mG)(fU)(mA)(fG)(mC)(fA)(mG)(fC)(mU)#(fG)#(mC)#(fU)#(mU)#(fC)#(mU)#(fC)	(fG)#(mC)#(fU)(mG)(fC)(mU)(fG)(mC)(fU)(mA)(fC)(mA)(fG)#(mA)#(fA)-TegChol			rs362273	2
SNP6-14	6	14	P(mU)#(fU)#(mC)(fU)(mG)(fU)(mA)(fG)(mC)(fA)(mG)(fC)(mA)#(fU)#(mC)#(fU)#(mU)#(fC)#(mU)#(fC)	(fG)#(mC)#(fU)(mG)(fC)(mU)(fG)(mC)(fU)(mA)(fC)(mA)(fG)#(mA)#(fA)-TegChol	30	>2000	rs362273	2
SNP6-15	6	15	P(mU)#(fU)#(mC)(fU)(mG)(fU)(mA)(fG)(mC)(fA)(mG)(fC)(mA)#(fG)#(mU)#(fU)#(mU)#(fC)#(mU)#(fC)	(fG)#(mC)#(fU)(mG)(fC)(mU)(fG)(mC)(fU)(mA)(fC)(mA)(fG)#(mA)#(fA)-TegChol			rs362273	2
SNP6-16	6	16	P(mU)#(fU)#(mC)(fU)(mG)(fU)(mA)(fG)(mC)(fA)(mG)(fC)(mA)#(fG)#(mC)#(fA)#(mU)#(fC)#(mU)#(fC)	(fG)#(mC)#(fU)(mG)(fC)(mU)(fG)(mC)(fU)(mA)(fC)(mA)(fG)#(mA)#(fA)-TegChol	18	333	rs362273	2
SNP7-0	7	0	P(mU)#(fA)#(mU)(fC)(mU)(fG)(mU)(fA)(mG)(fC)(mA)(fG)(mC)#(fA)#(mG)#(fC)#(mU)#(fU)#(mC)#(fU)	(fC)#(mU)#(fG)(mC)(fU)(mG)(fC)(mU)(fA)(mC)(fA)(mG)(fA)#(mU)#(fA)-TegChol			rs362273	1
SNP8-0	8	0	P(mU)#(fG)#(mA)(fU)(mC)(fU)(mG)(fU)(mA)(fG)(mC)(fA)(mG)#(fC)#(mA)#(fG)#(mC)#(fU)#(mU)#(fC)	(fU)#(mG)#(fC)(mU)(fG)(mC)(fU)(mA)(fC)(mA)(fG)(mA)(fU)#(mC)#(fA)-TegChol			rs362273	1
SNP9-0	9	0	P(mU)#(fU)#(mG)(fA)(mU)(fC)(mU)(fG)(mU)(fA)(mG)(fC)(mA)#(fG)#(mC)#(fA)#(mG)#(fC)#(mU)#(fU)	(fG)#(mC)#(fU)(mG)(fC)(mU)(fA)(mC)(fA)(mG)(fA)(mU)(fC)#(mA)#(fA)-TegChol			rs362273	1
SNP10-0	10	0	P(mU)#(fU)#(mU)(fG)(mA)(fU)(mC)(fU)(mG)(fU)(mA)(fG)(mC)#(fA)#(mG)#(fC)#(mA)#(fG)#(mC)#(fU)	(fC)#(mU)#(fG)(mC)(fU)(mA)(fC)(mA)(fG)(mA)(fU)(mC)(fA)#(mA)#(fA)-TegChol			rs362273	1
SNP11-0	11	0	P(mU)#(fG)#(mU)(fU)(mG)(fA)(mU)(fC)(mU)(fG)(mU)(fA)(mG)#(fC)#(mA)#(fG)#(mC)#(fA)#(mG)#(fC)	(fU)#(mG)#(fC)(mU)(fA)(mC)(fA)(mG)(fA)(mU)(fC)(mA)(fA)#(mC)#(fA)-TegChol			rs362273	1
SNP12-0	12	0	P(mU)#(fG)#(mG)(fU)(mU)(fG)(mA)(fU)(mC)(fU)(mG)(fU)(mA)#(fG)#(mC)#(fA)#(mG)#(fC)#(mA)#(fG)	(fG)#(mC)#(fU)(mA)(fC)(mA)(fG)(mA)(fU)(mC)(fA)(mA)(fC)#(mC)#(fA)-TegChol			rs362273	1
SNP13-0	13	0	P(mU)#(fG)#(mG)(fG)(mU)(fU)(mG)(fA)(mU)(fC)(mU)(fG)(mU)#(fA)#(mG)#(fC)#(mA)#(fG)#(mC)#(fA)	(fC)#(mU)#(fA)(mC)(fA)(mG)(fA)(mU)(fC)(mA)(fA)(mC)(fC)#(mC)#(fA)-TegChol			rs362273	1
SNP 6-f11	6	11	P(mU)#(fU)#(mC)(fU)(mG)(fU)(mA)(fG)(mC)(fA)(fU)(fC)(mA)#(fG)#(mC)#(fU)#(mU)#(fC)#(mU)#(fC)	(fG)#(mC)#(fU)(mG)(mC)(mU)(fG)(mC)(fU)(mA)(fC)(mA)(fG)#(mA)#(fA)-TegChol			rs362273	3
SNP f6-f11	6	11	P(mU)#(fU)#(mC)(fU)(fG)(fU)(fA)(fG)(mC)(fA)(fU)(fC)(mA)#(fG)#(mC)#(fU)#(mU)#(fC)#(mU)#(fC)	(fG)#(mC)#(fU)(mG)(mC)(mU)(fG)(mC)(mU)(mA)(mC)(mA)(fG)#(mA)#(fA)-TegChol			rs362273	3
SNP f6-m11	6	11	P(mU)#(fU)#(mC)(fU)(fG)(fU)(fA)(fG)(mC)(mA)(mU)(mC)(mA)#(fG)#(mC)#(fU)#(mU)#(fC)#(mU)#(fC)	(fG)#(mC)#(fU)(fG)(fC)(fU)(fG)(mC)(mU)(mA)(mC)(mA)(fG)#(mA)#(fA)-TegChol			rs362273	3
SNP m6-f11	6	11	P(mU)#(fU)#(mC)(fU)(mG)(mU)(mA)(fG)(mC)(fA)(fU)(fC)(mA)#(fG)#(mC)#(fU)#(mU)#(fC)#(mU)#(fC)	(fG)#(mC)#(fU)(mG)(mC)(mU)(fG)(mC)(fU)(fA)(fC)(mA)(fG)#(mA)#(fA)-TegChol			rs362273	3
SNP m6-m11	6	11	P(mU)#(fU)#(mC)(fU)(mG)(mU)(mA)(fG)(mC)(mA)(mU)(mC)(mA)#(fG)#(mC)#(fU)#(mU)#(fC)#(mU)#(fC)	(fG)#(mC)#(fU)(fG)(fC)(fU)(fG)(mC)(fU)(fA)(fC)(mA)(fG)#(mA)#(fA)-TegChol			rs362273	3
SNP 6-m11	6	11	P(mU)#(fU)#(mC)(fU)(mG)(fU)(mA)(fG)(mC)(mA)(mU)(mC)(mA)#(fG)#(mC)#(fU)#(mU)#(fC)#(mU)#(fC)	(fG)#(mC)#(fU)(fG)(fC)(fU)(fG)(mC)(fU)(mA)(fC)(mA)(fG)#(mA)#(fA)-TegChol			rs362273	3
SNP fm6-11	6	11	P(mU)#(fU)#(mC)(fU)(fG)(mU)(fA)(fG)(mC)(fA)(mU)(fC)(mA)#(fG)#(mC)#(fU)#(mU)#(fC)#(mU)#(fC)	(fG)#(mC)#(fU)(mG)(fC)(mU)(fG)(mC)(mU)(fA)(mC)(mA)(fG)#(mA)#(fA)-TegChol			rs362273	3
SNP f6-11	6	11	P(mU)#(fU)#(mC)(fU)(fG)(fU)(fA)(fG)(mC)(fA)(mU)(fC)(mA)#(fG)#(mC)#(fU)#(mU)#(fC)#(mU)#(fC)	(fG)#(mC)#(fU)(mG)(fC)(mU)(fG)(mC)(mU)(mA)(mC)(mA)(fG)#(mA)#(fA)-TegChol			rs362273	3
SNP m6-11	6	11	P(mU)#(fU)#(mC)(fU)(mG)(mU)(mA)(fG)(mC)(fA)(mU)(fC)(mA)#(fG)#(mC)#(fU)#(mU)#(fC)#(mU)#(fC)	(fG)#(mC)#(fU)(mG)(fC)(mU)(fG)(mC)(fU)(fA)(fC)(mA)(fG)#(mA)#(fA)-TegChol			rs362273	3
SNP2	2	0	P(mU)#(fG)#(mC)(fA)(mG)(fA)(mC)(fU)(mU)(fC)(mC)(fA)(mA)#(fA)#(mG)#(fG)#(mC)#(fU)#(mC)#(fC)	(fC)#(mU)#(fU)(mU)(fG)(mG)(fA)(mA)(fG)(mU)(fC)(mU)(fG)#(mC)#(fA)-TegChol			rs362307	S5
SNP3	3	0	P(mU)#(fC)#(mG)(fC)(mA)(fG)(mA)(fC)(mU)(fU)(mC)(fC)(mA)#(fA)#(mA)#(fG)#(mG)#(fC)#(mU)#(fC)	(fU)#(mU)#(fU)(mG)(fG)(mA)(fA)(mG)(fU)(mC)(fU)(mG)(fC)#(mG)#(fA)-TegChol	35	438	rs362307	S5
SNP4	4	0	P(mU)#(fG)#(mC)(fG)(mC)(fA)(mG)(fA)(mC)(fU)(mU)(fC)(mC)#(fA)#(mA)#(fA)#(mG)#(fG)#(mC)#(fU)	(fU)#(mU)#(fG)(mG)(fA)(mA)(fG)(mU)(fC)(mU)(fG)(mC)(fG)#(mC)#(fA)-TegChol			rs362307	S5
SNP5	5	0	P(mU)#(fG)#(mG)(fC)(mG)(fC)(mA)(fG)(mA)(fC)(mU)(fU)(mC)#(fC)#(mA)#(fA)#(mA)#(fG)#(mG)#(fC)	(fU)#(mG)#(fG)(mA)(fA)(mG)(fU)(mC)(fU)(mG)(fC)(mG)(fC)#(mC)#(fA)-TegChol			rs362307	S5
SNP6	6	0	P(mU)#(fG)#(mG)(fG)(mC)(fG)(mC)(fA)(mG)(fA)(mC)(fU)(mU)#(fC)#(mC)#(fA)#(mA)#(fA)#(mG)#(fG)	(fG)#(mG)#(fA)(mA)(fG)(mU)(fC)(mU)(fG)(mC)(fG)(mC)(fC)#(mC)#(fA)-TegChol			rs362307	S5
SNP7	7	0	P(mU)#(fA)#(mG)(fG)(mG)(fC)(mG)(fC)(mA)(fG)(mA)(fC)(mU)#(fU)#(mC)#(fC)#(mA)#(fA)#(mA)#(fG)	(fG)#(mA)#(fA)(mG)(fU)(mC)(fU)(mG)(fC)(mG)(fC)(mC)(fC)#(mU)#(fA)-TegChol			rs362307	S5
SNP8	8	0	P(mU)#(fA)#(mA)(fG)(mG)(fG)(mC)(fG)(mC)(fA)(mG)(fA)(mC)#(fU)#(mU)#(fC)#(mC)#(fA)#(mA)#(fA)	(fA)#(mA)#(fG)(mU)(fC)(mU)(fG)(mC)(fG)(mC)(fC)(mC)(fU)#(mU)#(fA)-TegChol			rs362307	S5
SNP9	9	0	P(mU)#(fC)#(mA)(fA)(mG)(fG)(mG)(fC)(mG)(fC)(mA)(fG)(mA)#(fC)#(mU)#(fU)#(mC)#(fC)#(mA)#(fA)	(fA)#(mG)#(fU)(mC)(fU)(mG)(fC)(mG)(fC)(mC)(fC)(mU)(fU)#(mG)#(fA)-TegChol			rs362307	S5
SNP10	10	0	P(mU)#(fA)#(mC)(fA)(mA)(fG)(mG)(fG)(mC)(fG)(mC)(fA)(mG)#(fA)#(mC)#(fU)#(mU)#(fC)#(mC)#(fA)	(fG)#(mU)#(fC)(mU)(fG)(mC)(fG)(mC)(fC)(mC)(fU)(mU)(fG)#(mU)#(fA)-TegChol			rs362307	S5
SNP11	11	0	P(mU)#(fC)#(mA)(fC)(mA)(fA)(mG)(fG)(mG)(fC)(mG)(fC)(mA)#(fG)#(mA)#(fC)#(mU)#(fU)#(mC)#(fC)	(fU)#(mC)#(fU)(mG)(fC)(mG)(fC)(mC)(fC)(mU)(fU)(mG)(fU)#(mG)#(fA)-TegChol			rs362307	S5
SNP12	12	0	P(mU)#(fG)#(mC)(fA)(mC)(fA)(mA)(fG)(mG)(fG)(mC)(fG)(mC)#(fA)#(mG)#(fA)#(mC)#(fU)#(mU)#(fC)	(fC)#(mU)#(fG)(mC)(fG)(mC)(fC)(mC)(fU)(mU)(fG)(mU)(fG)#(mC)#(fA)-TegChol			rs362307	S5
SNP13	13	0	P(mU)#(fG)#(mG)(fC)(mA)(fC)(mA)(fA)(mG)(fG)(mG)(fC)(mG)#(fC)#(mA)#(fG)#(mA)#(fC)#(mU)#(fU)	(fU)#(mG)#(fC)(mG)(fC)(mC)(fC)(mU)(fU)(mG)(fU)(mG)(fC)#(mC)#(fA)-TegChol			rs362307	S5
SNP 3-2	3	2	P(mU)#(fU)#(mG)(fC)(mA)(fG)(mA)(fC)(mU)(fU)(mC)(fC)(mA)#(fA)#(mA)#(fG)#(mG)#(fC)#(mU)#(fC)	(fU)#(mU)#(fU)(mG)(fG)(mA)(fA)(mG)(fU)(mC)(fU)(mG)(fC)#(mA)#(fA)-TegChol			rs362307	S5
SNP 3-4	3	4	P(mU)#(fC)#(mG)(fU)(mA)(fG)(mA)(fC)(mU)(fU)(mC)(fC)(mA)#(fA)#(mA)#(fG)#(mG)#(fC)#(mU)#(fC)	(fU)#(mU)#(fU)(mG)(fG)(mA)(fA)(mG)(fU)(mC)(fU)(mA)(fC)#(mG)#(fA)-TegChol			rs362307	S5
SNP 3-5C	3	5	P(mU)#(fC)#(mG)(fC)(mC)(fG)(mA)(fC)(mU)(fU)(mC)(fC)(mA)#(fA)#(mA)#(fG)#(mG)#(fC)#(mU)#(fC)	(fU)#(mU)#(fU)(mG)(fG)(mA)(fA)(mG)(fU)(mC)(fG)(mG)(fC)#(mG)#(fA)-TegChol	92	>1500	rs362307	S5
SNP 3-5G	3	5	P(mU)#(fC)#(mG)(fC)(mG)(fG)(mA)(fC)(mU)(fU)(mC)(fC)(mA)#(fA)#(mA)#(fG)#(mG)#(fC)#(mU)#(fC)	(fU)#(mU)#(fU)(mG)(fG)(mA)(fA)(mG)(fU)(mC)(fC)(mG)(fC)#(mG)#(fA)-TegChol			rs362307	S5
SNP 3-6	3	6	P(mU)#(fC)#(mG)(fC)(mA)(fU)(mA)(fC)(mU)(fU)(mC)(fC)(mA)#(fA)#(mA)#(fG)#(mG)#(fC)#(mU)#(fC)	(fU)#(mU)#(fU)(mG)(fG)(mA)(fA)(mG)(fU)(mA)(fU)(mG)(fC)#(mG)#(fA)-TegChol			rs362307	S5
SNP 3-7C	3	7	P(mU)#(fC)#(mG)(fC)(mA)(fG)(mC)(fC)(mU)(fU)(mC)(fC)(mA)#(fA)#(mA)#(fG)#(mG)#(fC)#(mU)#(fC)	(fU)#(mU)#(fU)(mG)(fG)(mA)(fA)(mG)(fG)(mC)(fU)(mG)(fC)#(mG)#(fA)-TegChol	400	>1500	rs362307	S5
SNP 3-7G	3	7	P(mU)#(fC)#(mG)(fC)(mA)(fG)(mG)(fC)(mU)(fU)(mC)(fC)(mA)#(fA)#(mA)#(fG)#(mG)#(fC)#(mU)#(fC)	(fU)#(mU)#(fU)(mG)(fG)(mA)(fA)(mG)(fC)(mC)(fU)(mG)(fC)#(mG)#(fA)-TegChol	20	83	rs362307	S5
SNP 3-8	3	8	P(mU)#(fC)#(mG)(fC)(mA)(fG)(mA)(fU)(mU)(fU)(mC)(fC)(mA)#(fA)#(mA)#(fG)#(mG)#(fC)#(mU)#(fC)	(fU)#(mU)#(fU)(mG)(fG)(mA)(fA)(mA)(fU)(mC)(fU)(mG)(fC)#(mG)#(fA)-TegChol	50	750	rs362307	S5
SNP 3-9	3	9	P(mU)#(fC)#(mG)(fC)(mA)(fG)(mA)(fC)(mA)(fU)(mC)(fC)(mA)#(fA)#(mA)#(fG)#(mG)#(fC)#(mU)#(fC)	(fU)#(mU)#(fU)(mG)(fG)(mA)(fU)(mG)(fU)(mC)(fU)(mG)(fC)#(mG)#(fA)-TegChol			rs362307	S5
SNP 3-10	3	10	P(mU)#(fC)#(mG)(fC)(mA)(fG)(mA)(fC)(mU)(fA)(mC)(fC)(mA)#(fA)#(mA)#(fG)#(mG)#(fC)#(mU)#(fC)	(fU)#(mU)#(fU)(mG)(fG)(mU)(fA)(mG)(fU)(mC)(fU)(mG)(fC)#(mG)#(fA)-TegChol			rs362307	S5
SNP 3-11	3	11	P(mU)#(fC)#(mG)(fC)(mA)(fG)(mA)(fC)(mU)(fU)(mU)(fC)(mA)#(fA)#(mA)#(fG)#(mG)#(fC)#(mU)#(fC)	(fU)#(mU)#(fU)(mG)(fA)(mA)(fA)(mG)(fU)(mC)(fU)(mG)(fC)#(mG)#(fA)-TegChol			rs362307	S5
SNP 3-12	3	12	P(mU)#(fC)#(mG)(fC)(mA)(fG)(mA)(fC)(mU)(fU)(mC)(fU)(mA)#(fA)#(mA)#(fG)#(mG)#(fC)#(mU)#(fC)	(fU)#(mU)#(fU)(mA)(fG)(mA)(fA)(mG)(fU)(mC)(fU)(mG)(fC)#(mG)#(fA)-TegChol			rs362307	S5
SNP 3-13	3	13	P(mU)#(fC)#(mG)(fC)(mA)(fG)(mA)(fC)(mU)(fU)(mC)(fC)(mC)#(fA)#(mA)#(fG)#(mG)#(fC)#(mU)#(fC)	(fU)#(mU)#(fG)(mG)(fG)(mA)(fA)(mG)(fU)(mC)(fU)(mG)(fC)#(mG)#(fA)-TegChol			rs362307	S5
SNP 3-13G	3	13	P(mU)#(fC)#(mG)(fC)(mA)(fG)(mA)(fC)(mU)(fU)(mC)(fC)(mG)#(fA)#(mA)#(fG)#(mG)#(fC)#(mU)#(fC)	(fU)#(mU)#(fC)(mG)(fG)(mA)(fA)(mG)(fU)(mC)(fU)(mG)(fC)#(mG)#(fA)-TegChol			rs362307	S5
SNP 3-14C	3	14	P(mU)#(fC)#(mG)(fC)(mA)(fG)(mA)(fC)(mU)(fU)(mC)(fC)(mA)#(fC)#(mA)#(fG)#(mG)#(fC)#(mU)#(fC)	(fU)#(mG)#(fU)(mG)(fG)(mA)(fA)(mG)(fU)(mC)(fU)(mG)(fC)#(mG)#(fA)-TegChol			rs362307	S5
SNP 3-14G	3	14	P(mU)#(fC)#(mG)(fC)(mA)(fG)(mA)(fC)(mU)(fU)(mC)(fC)(mA)#(fG)#(mA)#(fG)#(mG)#(fC)#(mU)#(fC)	(fU)#(mC)#(fU)(mG)(fG)(mA)(fA)(mG)(fU)(mC)(fU)(mG)(fC)#(mG)#(fA)-TegChol			rs362307	S5
SNP 3-15C	3	15	P(mU)#(fC)#(mG)(fC)(mA)(fG)(mA)(fC)(mU)(fU)(mC)(fC)(mA)#(fA)#(mC)#(fG)#(mG)#(fC)#(mU)#(fC)	(fG)#(mU)#(fU)(mG)(fG)(mA)(fA)(mG)(fU)(mC)(fU)(mG)(fC)#(mG)#(fA)-TegChol			rs362307	S5
SNP 3-15G	3	15	P(mU)#(fC)#(mG)(fC)(mA)(fG)(mA)(fC)(mU)(fU)(mC)(fC)(mA)#(fA)#(mG)#(fG)#(mG)#(fC)#(mU)#(fC)	(fC)#(mU)#(fU)(mG)(fG)(mA)(fA)(mG)(fU)(mC)(fU)(mG)(fC)#(mG)#(fA)-TegChol			rs362307	S5
SNP6-11(A) Dio	6	11	V(mU)#(fU)#(mC)(fU)(mG)(fU)(mA)(fG)(mC)(fA)(mU)(fC)(mA)#(fG)#(mC)#(fU)#(mU)#(fC)#(mU)#(fC)	(fG)#(mC)#(fU)(mG)(fA)(mU)(fG)(mC)(fU)(mA)(fC)(mA)(fG)#(mA)#(fA)-Dio			rs362273	5,S6,S7,S8

notation	meaning
P	phosphate group
V	vinylphosphonate
m	2'Ome
f	2'F
#	phosphorothioate
TegChol	teg linker linked to cholesterol
Dio	divalent siRNAs for in vivo use have a teg linker attached to an additional sense strand, no cholesterol.

Supplementary Table 2. Sequences used in the study—without chemical modifications.

siRNA ID	SNP position	additional mismatch position	ntisense strand sequen	sense strand sequence	IC 50 target [nM]	IC50 non-target [nM]	Target SNP site
SNP2-0	2	0	UUAGCAGCAGCUUCUCGUGG	AGAAGCUGCUGCUAA			rs362273
SNP2-3G	2	3	UUGGCAGCAGCUUCUCGUGG	AGAAGCUGCUGCUAA			rs362273
SNP2-3U	2	3	UUUGCAGCAGCUUCUCGUGG	AGAAGCUGCUGCUAA			rs362273
SNP2-3C	2	3	UUCGCAGCAGCUUCUCGUGG	AGAAGCUGCUGCUAA			rs362273
SNP2-4	2	4	UUAUCAGCAGCUUCUCGUGG	AGAAGCUGCUGCUAA			rs362273
SNP2-5	2	5	UUAGUAGCAGCUUCUCGUGG	AGAAGCUGCUGCUAA			rs362273
SNP2-6	2	6	UUAGCUGCAGCUUCUCGUGG	AGAAGCUGCUGCUAA			rs362273
SNP2-7	2	7	UUAGCAUCAGCUUCUCGUGG	AGAAGCUGCUGCUAA			rs362273
SNP2-8	2	8	UUAGCAGUAGCUUCUCGUGG	AGAAGCUGCUGCUAA			rs362273
SNP2-9	2	9	UUAGCAGCUGCUUCUCGUGG	AGAAGCUGCUGCUAA			rs362273
SNP2-10	2	10	UUAGCAGCAUCUUCUCGUGG	AGAAGCUGCUGCUAA			rs362273
SNP2-11	2	11	UUAGCAGCAGUUUCUCGUGG	AGAAGCUGCUGCUAA			rs362273
SNP2-12	2	12	UUAGCAGCAGCAUCUCGUGG	AGAAGCUGCUGCUAA			rs362273
SNP2-13	2	13	UUAGCAGCAGCUACUCGUGG	AGAAGCUGCUGCUAA			rs362273
SNP2-14	2	14	UUAGCAGCAGCUUUUCGUGG	AGAAGCUGCUGCUAA			rs362273
SNP2-15	2	15	UUAGCAGCAGCUUCACGUGG	AGAAGCUGCUGCUAA			rs362273
SNP2-16	2	16	UUAGCAGCAGCUUCUUGUGG	AGAAGCUGCUGCUAA			rs362273
SNP3-0	3	0	UGUAGCAGCAGCUUCUCGUG	GAAGCUGCUGCUACA			rs362273
SNP4-0	4	0	UUGUAGCAGCAGCUUCUCGU	AAGCUGCUGCUACAA	38	803	rs362273
SNP4-2	4	2	UAGUAGCAGCAGCUUCUCGU	AAGCUGCUGCUACAA			rs362273
SNP4-3U	4	3	UUUUAGCAGCAGCUUCUCGU	AAGCUGCUGCUACAA			rs362273
SNP4-3A	4	3	UUAUAGCAGCAGCUUCUCGU	AAGCUGCUGCUACAA			rs362273
SNP4-5C	4	5	UUGUCGCAGCAGCUUCUCGU	AAGCUGCUGCUACAA			rs362273
SNP4-5G	4	5	UUGUGGCAGCAGCUUCUCGU	AAGCUGCUGCUACAA			rs362273
SNP4-6	4	6	UUGUAUCAGCAGCUUCUCGU	AAGCUGCUGCUACAA			rs362273
SNP4-7	4	7	UUGUAGUAGCAGCUUCUCGU	AAGCUGCUGCUACAA	17	>5000	rs362273
SNP4-8	4	8	UUGUAGCUGCAGCUUCUCGU	AAGCUGCUGCUACAA	24	220	rs362273
SNP4-9	4	9	UUGUAGCAUCAGCUUCUCGU	AAGCUGCUGCUACAA			rs362273
SNP4-10	4	10	UUGUAGCAGUAGCUUCUCGU	AAGCUGCUGCUACAA			rs362273
SNP4-11	4	11	UUGUAGCAGCUGCUUCUCGU	AAGCUGCUGCUACAA			rs362273
SNP4-12	4	12	UUGUAGCAGCAUCUUCUCGU	AAGCUGCUGCUACAA			rs362273
SNP4-13	4	13	UUGUAGCAGCAGUUUCUCGU	AAGCUGCUGCUACAA			rs362273
SNP4-14	4	14	UUGUAGCAGCAGCAUCUCGU	AAGCUGCUGCUACAA			rs362273
SNP4-15	4	15	UUGUAGCAGCAGCUACUCGU	AAGCUGCUGCUACAA			rs362273
SNP4-16	4	16	UUGUAGCAGCAGCUUUUCGU	AAGCUGCUGCUACAA			rs362273
SNP5-0	5	0	UCUGUAGCAGCAGCUUCUCG	AGCUGCUGCUACAGA			rs362273
SNP6-0	6	0	UUCUGUAGCAGCAGCUUCUC	GCUGCUGCUACAGAA	24	174	rs362273
SNP6-2	6	2	UACUGUAGCAGCAGCUUCUC	GCUGCUGCUACAGAA			rs362273
SNP6-3	6	3	UUUUGUAGCAGCAGCUUCUC	GCUGCUGCUACAGAA			rs362273
SNP6-4	6	4	UUCAGUAGCAGCAGCUUCUC	GCUGCUGCUACAGAA			rs362273
SNP6-5U	6	5	UUCUUUAGCAGCAGCUUCUC	GCUGCUGCUACAGAA			rs362273
SNP6-5A	6	5	UUCUAUAGCAGCAGCUUCUC	GCUGCUGCUACAGAA			rs362273
SNP6-7C	6	7	UUCUGUCGCAGCAGCUUCUC	GCUGCUGCUACAGAA			rs362273
SNP6-7G	6	7	UUCUGUGGCAGCAGCUUCUC	GCUGCUGCUACAGAA			rs362273
SNP6-8	6	8	UUCUGUAUCAGCAGCUUCUC	GCUGCUGCUACAGAA			rs362273
SNP6-9	6	9	UUCUGUAGUAGCAGCUUCUC	GCUGCUGCUACAGAA			rs362273
SNP6-10	6	10	UUCUGUAGCUGCAGCUUCUC	GCUGCUGCUACAGAA			rs362273
SNP6-11 (G)	6	11	UUCUGCAGCAUCAGCUUCUC	GCUGCUGCUGCAGAA			rs362273
SNP6-11 (A)	6	11	UUCUGUAGCAUCAGCUUCUC	GCUGCUGCUACAGAA	28	>4500	rs362273
SNP6-12	6	12	UUCUGUAGCAGUAGCUUCUC	GCUGCUGCUACAGAA	24	>1000	rs362273
SNP6-13	6	13	UUCUGUAGCAGCUGCUUCUC	GCUGCUGCUACAGAA			rs362273
SNP6-14	6	14	UUCUGUAGCAGCAUCUUCUC	GCUGCUGCUACAGAA	30	>2000	rs362273
SNP6-15	6	15	UUCUGUAGCAGCAGUUUCUC	GCUGCUGCUACAGAA			rs362273
SNP6-16	6	16	UUCUGUAGCAGCAGCAUCUC	GCUGCUGCUACAGAA	18	333	rs362273
SNP7-0	7	0	UAUCUGUAGCAGCAGCUUCU	CUGCUGCUACAGAUA			rs362273
SNP8-0	8	0	UGAUCUGUAGCAGCAGCUUC	UGCUGCUACAGAUCA			rs362273
SNP9-0	9	0	UUGAUCUGUAGCAGCAGCUU	GCUGCUACAGAUCAA			rs362273
SNP10-0	10	0	UUUGAUCUGUAGCAGCAGCU	CUGCUACAGAUCAAA			rs362273
SNP11-0	11	0	UGUUGAUCUGUAGCAGCAGC	UGCUACAGAUCAACA			rs362273
SNP12-0	12	0	UGGUUGAUCUGUAGCAGCAG	GCUACAGAUCAACCA			rs362273
SNP13-0	13	0	UGGGUUGAUCUGUAGCAGCA	CUACAGAUCAACCCA			rs362273
SNP2	2	0	UGCAGACUUCCAAAGGCUCC	CUUUGGAAGUCUGCA			rs362307
SNP3	3	0	UCGCAGACUUCCAAAGGCUC	UUUGGAAGUCUGCGA	35	438	rs362307
SNP4	4	0	UGCGCAGACUUCCAAAGGCU	UUGGAAGUCUGCGCA			rs362307
SNP5	5	0	UGGCGCAGACUUCCAAAGGC	UGGAAGUCUGCGCCA			rs362307
SNP6	6	0	UGGGCGCAGACUUCCAAAGG	GGAAGUCUGCGCCCA			rs362307
SNP7	7	0	UAGGGCGCAGACUUCCAAAG	GAAGUCUGCGCCCUA			rs362307
SNP8	8	0	UAAGGGCGCAGACUUCCAAA	AAGUCUGCGCCCUUA			rs362307
SNP9	9	0	UCAAGGGCGCAGACUUCCAA	AGUCUGCGCCCUUGA			rs362307
SNP10	10	0	UACAAGGGCGCAGACUUCCA	GUCUGCGCCCUUGUA			rs362307
SNP11	11	0	UCACAAGGGCGCAGACUUCC	UCUGCGCCCUUGUGA			rs362307
SNP12	12	0	UGCACAAGGGCGCAGACUUC	CUGCGCCCUUGUGCA			rs362307
SNP13	13	0	UGGCACAAGGGCGCAGACUU	UGCGCCCUUGUGCCA			rs362307
SNP 3-2	3	2	UUGCAGACUUCCAAAGGCUC	UUUGGAAGUCUGCAA			rs362307
SNP 3-4	3	4	UCGUAGACUUCCAAAGGCUC	UUUGGAAGUCUACGA			rs362307
SNP 3-5C	3	5	UCGCCGACUUCCAAAGGCUC	UUUGGAAGUCGGCGA	>1500	92	rs362307
SNP 3-5G	3	5	UCGCGGACUUCCAAAGGCUC	UUUGGAAGUCCGCGA			rs362307
SNP 3-6	3	6	UCGCAUACUUCCAAAGGCUC	UUUGGAAGUAUGCGA			rs362307
SNP 3-7C	3	7	UCGCAGCCUUCCAAAGGCUC	UUUGGAAGGCUGCGA	400	1500	rs362307
SNP 3-7G	3	7	UCGCAGGCUUCCAAAGGCUC	UUUGGAAGCCUGCGA	20	83	rs362307
SNP 3-8	3	8	UCGCAGAUUUCCAAAGGCUC	UUUGGAAAUCUGCGA	20	750	rs362307
SNP 3-9	3	9	UCGCAGACAUCCAAAGGCUC	UUUGGAUGUCUGCGA			rs362307
SNP 3-10	3	10	UCGCAGACUACCAAAGGCUC	UUUGGUAGUCUGCGA			rs362307
SNP 3-11	3	11	UCGCAGACUUUCAAAGGCUC	UUUGAAAGUCUGCGA			rs362307
SNP 3-12	3	12	UCGCAGACUUCUAAAGGCUC	UUUAGAAGUCUGCGA			rs362307
SNP 3-13	3	13	UCGCAGACUUCCCAAGGCUC	UUGGGAAGUCUGCGA			rs362307
SNP 3-13G	3	13	UCGCAGACUUCCGAAGGCUC	UUCGGAAGUCUGCGA			rs362307
SNP 3-14C	3	14	UCGCAGACUUCCACAGGCUC	UGUGGAAGUCUGCGA			rs362307
SNP 3-14G	3	14	UCGCAGACUUCCAGAGGCUC	UCUGGAAGUCUGCGA			rs362307
SNP 3-15C	3	15	UCGCAGACUUCCAACGGCUC	GUUGGAAGUCUGCGA			rs362307
SNP 3-15G	3	15	UCGCAGACUUCCAAGGGCUC	CUUGGAAGUCUGCGA			rs362307
