# Supplementary material for: Chemical engineering of therapeutic siRNAs for allele-specific gene silencing in Huntington’s disease models
Source: Nat Commun. 2022 Oct 3;13:5802. doi: 10.1038/s41467-022-33061-x (PMC9530163; doi:10.1038/s41467-022-33061-x)
Supplement: Supplementary file 3 — Reporting Summary [file 41467_2022_33061_MOESM3_ESM.pdf]

nature portfolio

Corresponding author(s): Anastasia Khvorova, Edith Pfister

Last updated by author(s): Aug 29, 2022

Reporting Summary

Nature Portfolio wishes to improve the reproducibility of the work that we publish. This form provides structure for consistency and transparency in reporting. For further information on Nature Portfolio policies, see our Editorial Policies and the Editorial Policy Checklist.

Statistics

For all statistical analyses, confirm that the following items are present in the figure legend, table legend, main text, or Methods section.

n/a	Confirmed	
☐	☒	The exact sample size (*n*) for each experimental group/condition, given as a discrete number and unit of measurement
☐	☒	A statement on whether measurements were taken from distinct samples or whether the same sample was measured repeatedly
☐	☒	The statistical test(s) used AND whether they are one- or two-sided<br>*Only common tests should be described solely by name; describe more complex techniques in the Methods section.*
☒	☐	A description of all covariates tested
☐	☒	A description of any assumptions or corrections, such as tests of normality and adjustment for multiple comparisons
☐	☒	A full description of the statistical parameters including central tendency (e.g. means) or other basic estimates (e.g. regression coefficient) AND variation (e.g. standard deviation) or associated estimates of uncertainty (e.g. confidence intervals)
☐	☒	For null hypothesis testing, the test statistic (e.g. *F*, *t*, *r*) with confidence intervals, effect sizes, degrees of freedom and *P* value noted<br>*Give P values as exact values whenever suitable.*
☒	☐	For Bayesian analysis, information on the choice of priors and Markov chain Monte Carlo settings
☒	☐	For hierarchical and complex designs, identification of the appropriate level for tests and full reporting of outcomes
☒	☐	Estimates of effect sizes (e.g. Cohen's *d*, Pearson's *r*), indicating how they were calculated

Our web collection on statistics for biologists contains articles on many of the points above.

Software and code

Policy information about availability of computer code

Data collection	No software was used
Data analysis	GraphPad Prism v. 9.0.0 was used for all statistical analyses, ImageJ v. 1.53s was used for image analysis

For manuscripts utilizing custom algorithms or software that are central to the research but not yet described in published literature, software must be made available to editors and reviewers. We strongly encourage code deposition in a community repository (e.g. GitHub). See the Nature Portfolio guidelines for submitting code & software for further information.

Data

Policy information about availability of data

All manuscripts must include a data availability statement. This statement should provide the following information, where applicable:

- Accession codes, unique identifiers, or web links for publicly available datasets
- A description of any restrictions on data availability
- For clinical datasets or third party data, please ensure that the statement adheres to our policy

Source data are provided with this paper.

Human research participants

Policy information about studies involving human research participants and Sex and Gender in Research.

Reporting on sex and gender	N/A
Population characteristics	N/A
Recruitment	N/A
Ethics oversight	N/A

Note that full information on the approval of the study protocol must also be provided in the manuscript.

Field-specific reporting

Please select the one below that is the best fit for your research. If you are not sure, read the appropriate sections before making your selection.

[x] Life sciences [ ] Behavioural & social sciences [ ] Ecological, evolutionary & environmental sciences

For a reference copy of the document with all sections, see nature.com/documents/nr-reporting-summary-flat.pdf

Life sciences study design

All studies must disclose on these points even when the disclosure is negative.

Sample size	Mice are useful for RNAi-based therapeutic studies since they model human physiology and pathology well enough to relate our observations to human neurodegeneration and other diseases for which the Khvorova laboratory designs siRNAs for. We have previously established and published that n= 6-10 per group produces robust data. An estimate of these numbers for an in vivo study are as follows: 4 compounds (target and controls) * 10 animals per treatment = 40 animals total.
Data exclusions	For protein data, data were excluded if there was no signal for the housekeeping gene.
Replication	In vivo experiments have subsequently been repeated in this and other mouse models and have been replicated. Experiments in NSCs were performed twice and were replicated.
Randomization	Mice were assigned to experimental groups randomly.
Blinding	Experimenters were not blinded.

Reporting for specific materials, systems and methods

We require information from authors about some types of materials, experimental systems and methods used in many studies. Here, indicate whether each material, system or method listed is relevant to your study. If you are not sure if a list item applies to your research, read the appropriate section before selecting a response.

Materials & experimental systems

n/a	Involved in the study	
[ ]	[x]	Antibodies
[ ]	[x]	Eukaryotic cell lines
[x]	[ ]	Palaeontology and archaeology
[ ]	[x]	Animals and other organisms
[x]	[ ]	Clinical data
[x]	[ ]	Dual use research of concern

Methods

n/a	Involved in the study	
[x]	[ ]	ChIP-seq
[x]	[ ]	Flow cytometry
[x]	[ ]	MRI-based neuroimaging

Antibodies

Antibodies used	HTT (aa1-17, Ab1, described in reference 75, obtained from the stock of the laboratory of Marian DiFiglia), GFAP (Millipore; Cat# AB5804, Lot# 3538088), DARPP32 (abcam; Cat# ab40801, Clone EP720Y, Lot# GR3213231-12),Vinculin (Sigma; Cat# V9131, Clone hVIN-1, Lot# 036M4797V) and GAPDH (Millipore; Cat# MAB374, Clone 6C5, Lot# 3527693), Iba1 (FujiFilm; Cat# 019-19741, Lot# CAG5175)

Validation	Primary antibodies have been validated by SDS-PAGE and western blot on mouse brain tissue and human cell lysates in the DiFiglia laboratory at Massachusetts General Hospital and produce a single strong band at the expected molecular weight. The manufacturers' validations statements for each antibody are as follows:<br>GFAP, "Routinely evaluated by immunohistochemistry on brain tissue, astrocytes and neurons. Species cross-reactivity Human, rat, bovine and canine. It is expected that the antibody will react with most mammalian GFAP due to homology."<br>DARPP32, "Positive control Rat brain lysate and human breast adenocarcinoma. Species reactivity: Mouse, rat, human, pig."<br>Vinculin, "The antibody reacts with the 116 kD vinculin band by immunoblotting. Good reactivity is obtained with human, bovine, chicken, dog, rat, mouse, turkey and xenopus."<br>GAPDH, "Evaluated by western blot on A431 lysates. Species cross-reactivity Human, porcine, canine, rat, mouse, rabbit, cat, and fish skeletal muscle. It has been reported that this antibody does not react with GAPDH from E. coli."<br>Iba1, "Specific to microglia and macrophage but not cross-reactive with neuron and astrocyte. Reactive with human, mouse, and rat Iba1."

Eukaryotic cell lines

Policy information about cell lines and Sex and Gender in Research

Cell line source(s)	HeLa (ATCC CCL-2). Human induced pluripotent stem cells (iPSCs) CS09iCTR-109n4 (CS vial ID: 1034860) and CS09iCTR-109n5 (CS vial ID: 1034589) hereafter referred as HD109 (clone n4 and n5) described by (Mattis VB et al., 2015)73, were acquired from Cedars-Sinai Regenerative Medicine Institute Induced Pluripotent Stem Cell Core. Experiments were performed with oversight of Human Embryonic Stem Cell Research Oversight Committee (ESCRO Committee) through the Partners (now MGB) Institutional Biosafety Committee (PIBC) at Partners Healthcare (ESCRO#: 2015-01-02 and PIBC Reg# 2017B000023).
Authentication	Human iPSCs were karyotyped after in-house expansion by Cell Line Genetics using array comparative genomic hybridization (aCGH, Agilent 60K Standard).
Mycoplasma contamination	No contamination detected
Commonly misidentified lines<br>(See ICLAC register)	No commonly misidentified lines were used in this study

Animals and other research organisms

Policy information about studies involving animals; ARRIVE guidelines recommended for reporting animal research, and Sex and Gender in Research

Laboratory animals	Mice: BACHD (Tg(HTT*97Q)IXwy) 6-8 weeks old. Mice were housed with a maximum of 5 per cage in a pathogen-free facility under standard conditions with access to food, water, and enrichment ad libitum. BAC97 male mice were bred to wild-type FVB female mice to produce mixed litters with ~50% heterozygous mutant offspring. The litters were weaned from their mothers at 18-21 days of age. Ear punches taken at the time of weaning were DNA extracted and used for genotyping by PCR and gel electrophoresis. Animals with confirmation of the BAC97 transgene were aged to 8 weeks for intracranial injection surgeries.
Wild animals	The study did not involve wild animals
Reporting on sex	Previous studies have not indicated sex-based differences in silencing therefore we did not consider this in the study design.
Field-collected samples	No field-collected samples were used in this study
Ethics oversight	All experimental studies involving animals were approved by the University of Massachusetts Medical School Institutional Animal Care and Use Committee (IACUC Protocol numbers A-978, A-2411) and performed according to the guidelines and regulations therein described. Stem cell experiments were performed with oversight of Human Embryonic Stem Cell Research Oversight Committee (ESCRO Committee) through the Partners (now MGB) Institutional Biosafety Committee (PIBC) at Partners Healthcare (ESCRO#: 2015-01-02 and PIBC Reg# 2017B000023).

Note that full information on the approval of the study protocol must also be provided in the manuscript.
